# Supplementary material for: Stacking Model‐Based Classifiers for Dealing With Multiple Sets of Noisy Labels
Source: Biom J. 2025 Mar 12;67(2):e70042. doi: 10.1002/bimj.70042 (PMC11898607; doi:10.1002/bimj.70042)
Supplement: Supplementary file 1 — Supporting Information [file BIMJ-67-e70042-s001.zip › density-based-ensemble-model-main/competitors_real_data/netlab-master/netlab-master/help/gmmprob.htm]

Netlab Reference Manual gmmprob


# gmmprob

## Purpose

Computes the data probability for a Gaussian mixture model.

## Synopsis

```
function prob = gmmprob(mix, x)
```

## Description

This function computes the unconditional data
density `p(x)` for a Gaussian mixture model. The data structure
`mix` defines the mixture model, while the matrix `x` contains
the data vectors. Each row of `x` represents a single vector.

## See Also

`gmm`, `gmmpost`, `gmmactiv`

---

**Pages:**
Index


---

Copyright (c) Ian T Nabney (1996-9)
